# Perioperative respiratory adverse events during ambulatory anesthesia in obese children

**DOI:** 10.1007/s11845-021-02659-3

**Published:** 2021-06-05

**Authors:** Vesna Marjanovic, Ivana Budic, Mladjan Golubovic, Christian Breschan

**Affiliations:** 1 Faculty of Medicine, University of Nis, Blvd. dr Zorana Djindjica 81, 18000 Nis, Serbia; 2 Clinic for Anesthesia and Intensive Therapy, Clinical Centre Nis, Blvd. dr Zorana Djindjica 48, 18000 Nis, Serbia; 3 Department of Anesthesia, Klinikum Klagenfurt, Feschigstrasse 11, 9020 Klagenfurt, Austria

**Keywords:** Anesthesia, Children, Day surgery, Obesity, Respiratory complications

## Abstract

Obesity is one of the most common clinical conditions in the pediatric population with an increasing prevalence ranging from 20 to 30% worldwide. It is well known that during ambulatory anesthesia, obese children are more prone to develop perioperative respiratory adverse events (PRAEs) associated with obesity. To avoid or at least minimize these adverse effects, a thorough preoperative assessment should be undertaken as well as consideration of specific anesthetic approaches such as preoxygenation before induction of anesthesia and optimizing drug dosing. The use of short-acting opioid and nonopioid analgesics and the frequent implementation of regional anesthesia should also be included. Noninvasive airway management, protective mechanical ventilation, and complete reversion of neuromuscular blockade and awake extubation also proved to be beneficial in preventing PRAEs. During the postoperative period, continuous monitoring of oxygenation and ventilation is mandatory in obese children. In the current review, we sought to provide recommendations that might help to reduce the severity of perioperative respiratory adverse events in obese children, which could be of particular importance for reducing the rate of unplanned hospitalizations and ultimately improving the overall postoperative recovery.

## Introduction

Obesity is the most common clinical condition in children with a worldwide prevalence ranging from 20 to 30% [1]. Anesthetic practices have shown that obese children are more prone to develop perioperative respiratory adverse events (PRAEs) that can cause critical incidents during ambulatory anesthesia [2]. The most common PRAEs in obese children undergoing elective general surgery include oxygen desaturation, difficult mask ventilation, airway obstruction, and bronchospasm, especially at younger ages [3]. The appearance of PRAEs is associated with prolonged hospital stay after surgery, increased hospital cost, and indirect costs among pediatric outpatients [4]. For these reasons given above, obesity poses an important challenge for performing ambulatory anesthesia.

The focus of this review is on the pathophysiological characteristics and comorbidities associated with obesity as well as the assessment of the preoperative risk in obese children undergoing ambulatory anesthesia. It is also necessary to evaluate different anesthetic techniques and their impact on the postoperative recovery of such patients. This information may help to prevent the development of severe respiratory adverse events and improve the overall postoperative recovery in these children. Due to limited information related only to obese children and outpatient anesthesia, many of the issues that are addressed by this report could be applied broadly to pediatric population as well for all types of surgeries than ambulatory surgeries alone.

### Pathophysiological characteristics and comorbidities

Obesity is defined on the basis of body mass index (BMI) which takes into account a child’s gender and age. Therefore, age- and sex-specific BMI must be interpreted by percentiles relative to other children of the same age and sex. In adults, a body mass index (BMI: weight/height^2^) over 25 and 30 kg/m^2^ delineates overweight and obesity, respectively. Childhood overweight according to the WHO is defined as BMI >  + 2SD, which corresponds to more extreme 98th percentile [5]. American definitions of childhood obesity designates a BMI ≥ 85th percentile as overweight, a BMI ≥ 95th percentile as obesity, and a BMI > 99th percentile as a morbid obesity [1, 6]. The estimated degree of childhood obesity depends on the definition used.

Whatever definition is used, obesity in children is predominantly associated with changes in various organ systems leading to comorbidities. In terms of the respiratory system, this primarily relates to difficulties in maintaining airway patency including difficult intubation and ventilation resulting in more frequently observed PRAEs as compared to normal weight children [7]. Deposition of adipose tissue in the upper airway may increase the tendency of the pharynx to collapse. In addition, a short and thick neck limits the extension during laryngoscopy which in combination with a larger tongue often results in a troublesome intubation. As a consequence of the upper airway obstruction, anesthesiologists may also encounter difficult mask ventilation [8], difficult laryngoscopy and intubation (more than one attempt), low oxygen saturation, and increased postoperative airway obstruction, such as laryngospasm [9]. The occurrence of perioperative laryngospasm is particularly frequent in obese children who suffer from sleep disordered breathing [10].Furthermore, childhood obesity is correlated with a reduction of functional residual capacity, expiratory reserve volume, forced expiratory volume in the first second, and diffusion capacity. A resulting high closing volume may cause atelectasis, air trapping, and intrapulmonary right to left shunting with possible hypoxemia. These changes are emphasized in the supine position when the abdominal pressure on the diaphragm is the highest [1]. Therefore, these patients require intraoperative mechanical ventilation modes which have been adjusted to their pulmonary physiology.

Development of comorbidities such as asthma and respiratory tract infections tends to be more frequent in obese than in normal-weight children [7]. Recent data has shown that the risk of developing childhood asthma, bronchial hyperreactivity, and obstructive sleep apnea is significantly increased in children who are overweight or obese [9, 11–13].

One comorbidity with high impact on the behavior of obese patients undergoing anesthesia is obstructive sleep apnea (OSA). Sleep apnea is reported in up to 80% of obese children and adolescents [14]. The presence of OSA in pediatric patients is suspected in cases with a positive family history or when symptoms such as snoring, daytime somnolence, learning disabilities, and apneic episode are present [15]. The gold standard for the diagnosis and evaluation of OSA is polysomnography as assessed by the apnea hypopnea index, intervals and nadir of arterial oxygen desaturation, and peak end-tidal CO_2_. In particular, the higher overnight end-tidal CO_2_ is indicative of hypoventilation [16]. The majority of obese children with OSA are at a higher risk for airway obstruction, diminished ventilatory response to CO_2_, and increased sensitivity to narcotic analgesics which primarily presents as a prolonged postoperative respiratory depression caused by opioids as compared to other children [17]. This exposes them to a higher risk of adverse respiratory events.

Cardiovascular comorbidities have also been frequently associated with childhood obesity due to an increased blood and stroke volume and consequently due to an increased cardiac output. Furthermore, arterial hypertension correlates positively with an increased BMI reaching an incidence of 20–30% in the obese pediatric population [18]. Left ventricular dysfunction has been reported in children affected with OSA as a consequence of long-lasting significantly decreased oxygen desaturations dropping at times to 70% during sleep [19]. Prolonged hypoxemia during daytime and nocturnal desaturation are associated with the development of pulmonary hypertension and cor pulmonale [20].

Among the most common endocrine, gastrointestinal and neurologic comorbidities are type 2 diabetes mellitus, gastroesophageal reflux, dyslipidemia, steatohepatitis, and pseudotumor cerebri. The severity of these comorbidities typically increases with the severity of obesity [21]. Obese children are at an increased risk of hyperinsulinemia, insulin resistance, prediabetes, and subsequently type 2 diabetes mellitus [22–24]. If type 2 diabetes mellitus appears during adolescence, deterioration of glycemic control and diabetes-related complications such as microalbuminuria, dyslipidemia and hypertension progress more rapidly than in those with the development of the same type of diabetes later in life [25]. In addition, obese patients often present with dysregulated immune responses to microorganisms and increased susceptibility to infections caused by the increased production of hormones, leptins, cytokines and other proinflammatory agents in adipocytes [26]. The data concerning the gastroesophageal reflux in obese children are controversial. One of the study showed increased incidence of gastroesophageal reflux in obese children, while the retrospective study by Elitsur and colleagues (2009) indicated no difference in gastroesophageal reflux between normal-weight, overweight, and obese children. Only independent risk factor for gastroesophageal reflux was in males [27, 28]. Obese children often have a higher risk of idiopathic intracranial hypertension (pseudotumor cerebri) with the symptoms like headache, vomiting, retro-ocular eye pain, and visual loss [29].

### Preoperative assessment

Successful management of obese children for ambulatory anesthesia involves thorough a preoperative assessment in order to predict potential difficulties during ambulatory anesthesia.

Physical examination of the upper airway, which includes the assessment of head and neck mobility, jaw mobility, mouth opening, inspection of oropharynx, and dentation, is needed because of potential difficulties in mask ventilation and endotracheal intubation. It is also important to obtain information about difficulties with previous ventilations/intubations and newly developed symptoms and signs of the upper airway obstruction. In obese children with asthma, the severity of asthma, current medication regimen, hospital visits, allergies, tolerance to physical activity, and previous anesthetic history should be established before anesthesia [30]. All patients with asthma should maintain their regular medications on the day of surgery. Developing acute respiratory tract infections may increase the existing airway hyperreactivity and obstruction and can provoke additional perioperative desaturation. Under these circumstances, elective surgical procedures need to be postponed for at least 2 weeks after respiratory tract infection until completing entire resolution of airway hyperreactivity [31]. Preoperative assessment of the existing symptoms and current management of obese children with OSA is important in predicting obstructive events that can occur postoperatively [32]. During preoperative period, obese children, with such respiratory comorbidities, should undergo pulmonary function tests and clinical evaluation done both by pulmonologists and anesthesiologists [30]. Any suspicion of cardiac disease requires clinical evaluation by cardiologist and electrocardiogram and echocardiography. In the case of hypertension, blood pressure should be measured prior to the procedure and a detailed history of the physical ability which correlates with the physical performance of obese children should be made. Thus, the degree of cardiopulmonary compromise can be estimated [33]. In diabetic obese children suffering from type 2 diabetes, preoperative glycemic control is mandatory [34]. Since there are no clear data concerning the management of probable gastroesophageal reflux in obese children, the management of these patients should be similar to the one performed in nonobese patients. Thus, fasting with forbiddance of clear liquids intake 2 h before surgery should be ensured [35].

Preoperative anxiety in obese patients can be reduced with midazolam premedication. However, sedative premedication in obese children must be performed cautiously due to the risk of significant respiratory depression, airway obstruction, and pulmonary atelectasis with consequent carbon dioxide retention and severe oxygen desaturation. Therefore, close oxyhemoglobin saturation monitoring by pulse oximetry should be mandatory after a sedative premedication preoperatively and of course as well during the recovery period [34]. Currently, sedation with dexmedetomidine is used as a superior sedative for premedication of children. In contrast to midazolam, higher doses of this medication do not cause airway obstruction or airway collapse [36, 37].

Intravenous access in obese children can be difficult and is more likely to fail at the first cannulation attempt than in normal-weight children [38]. Therefore, obese children may require ultrasound-guided cannulation in order to find the proper peripheral or central venous route.

### Intraoperative management

An important difficulty in obese pediatric patients is calculating the optimal drug doses required for induction and maintenance of anesthesia. Childhood obesity is associated with physiological and pharmacological changes that affect drug absorption, metabolism, and elimination [39]. The lack of evidence-based guidelines and small number of pharmacokinetic studies in obese children necessitates extrapolation from studies in adults. Conclusions cannot be made straight-forward due to physiological differences between children and adults in body composition and renal/hepatic metabolism.

Calculation of the drug doses are based on the patient’s total body weight (TBW), ideal body weight (IBW), and lean body weight (LBW). Absolute LBW is increased in obese children by approximately 20–40% as compared to normal-weight children [40]. Lean body weight can be estimated by the following formula: LBW = IBW + 0.3(TBW − IBW). Drug dosing is generally based on the volume of distribution for the loading dose and on the clearance for maintenance [41]. Thus, the lipophilic drugs have a higher volume of distribution than hydrophilic drugs as they distribute in lean and fat tissues. Hydrophilic drugs show very little change in volume distribution, since their distribution is limited to lean tissues only. Therefore, the dose of lipophilic drugs should be calculated based on the TBW, while hydrophilic drugs should be calculated according to IBW in obese children [39, 42]. On the other hand, dose determination of highly lipophilic anesthetics based on TBW can lead to overdose with consequential risk for the development of adverse effects [43]. Overdosing can be avoided by titrating lipophilic anesthetics using a dose estimation formula for LBW and by monitoring the depth of anesthesia in obese children [43, 44]. Also, drug clearance might be altered in obese individuals whose hepatic and renal clearance may be increased in correlation with LBW [41]. To date, there is no solid evidence that increased hepatic activity in obese children could change anesthetic clearance. Details concerning individual dosing of commonly used anesthetics are extensively given elsewhere [45, 46].

There are several short acting anesthetics suitable for ambulatory anesthesia, such as propofol, sevoflurane, and desflurane, with a lower incidence of PRAEs [47, 48]. If the age of the child permits the placement of an intravenous cannula, intravenous anesthetics are preferable for the induction to anesthesia. In cases of unsuccessful placement of an intravenous cannula, inhalation induction is recommended for obese children [47].

Among intravenous anesthetics, propofol is recommended for induction and maintenance of anesthesia in obese children. Propofol is a highly lipophilic drug. Therefore, obese children require a lower induction dose of propofol, which should be calculated based on LBW and not TBW to achieve the same clinical effects as in normal-weight children [49]. The obese children require 2.0 mg/kg for induction of anesthesia in comparison with 3.2 mg/kg for nonobese patients [49, 50]. In case of morbidly obese children, the dosing of propofol for maintenance of anesthesia has to change. One study suggests the dosage of propofol for these patients should be based on TBW using an allometric function [51]. However, when the maintenance doses of propofol are calculated based on the adjusted body weight, there are the increased incidence of somnolence for the first 30 min of recovery from anesthesia and postoperative respiratory adverse events. Until better weight-appropriate dosing regimens have been established, the titration of the propofol dose guided by targeted bispectral index (BIS) levels could minimize relative overdosing and its consequences [44]. One should be careful with the use of BIS in infants, due to age differences in BIS [52].

Sevoflurane and desflurane are the most commonly used inhalational agents for the induction of anesthesia in the pediatric population. Sevoflurane provides better hemodynamic stability and less airway irritability as compared to desflurane. For these reasons, desflurane usage is restricted and sevoflurane is recommended as the first-choice inhalational induction agent in obese children [53]. On the other hand, desflurane has a lower lipid solubility; it provides faster recovery from anesthesia and earlier discharge of patients [54, 55], which makes this anesthetic more attractive for maintenance of anesthesia than sevoflurane [56]. Despite that, anesthesiologists should be cautious using desflurane for maintenance of anesthesia in children due to possible glottic stenosis or desaturation events [53, 57]. Unfortunately, previous studies have been performed only on normal-weight children, demanding more studies in obese children to determine the benefit of desflurane use in maintaining of anesthesia.

Adequate pain control in obese children during surgical procedures is mainly achieved by opioid analgesics. Short-acting opioids are preferable in comparison to long-acting ones considering the faster recovery of pharyngeal tone and prevention of postoperative respiratory depression with hypoxemia and hypercarbia. With respect to the pharmacokinetic profile, remifentanil and alfentanil are attractive options for intraoperative analgesia with minimal postoperative residual effects [58]. Pharmacokinetic of remifentanil, as a hydrophilic drug, is unaltered in obese versus normal-weight subjects. Therefore, remifentanil pharmacokinetics are more closely related to LBW than to TBW, in order to avoid the risk of its side effects. Clinically, this means that remifentanil dosing regimens should be based on IBW or LBW and not on TBW [59, 60]. Alfentanil is less lipophilic opioid than fentanyl. However, in the absence of new data examining the effects of obesity on pharmacokinetic of alfentanil, both LBW and TBW have been suggested as dosing scalars for alfentanil [45]. Morphine as a hydrophilic opioid requires loading and maintenance dosing based on IBW [46, 59]. Yet, this approach in obese children may cause overdose [43]. In addition, children with OSA and previous recurrent hypoxemia may have increased analgesic sensitivity to subsequent morphine administration and consequential postoperative respiratory adverse events [61, 62]. Therefore, great caution is required when determining the dose of opioids for these patients.

The numerous disadvantages of opioid analgesics urge the continuous search for more desirable analgesics with fewer unwanted effects. Thus, a multimodal approach to analgesia with the co-administration of nonopioid adjuvant analgesic agents and regional anesthesia is recommended. The intraoperative use of these technics have reduced the need of intraoperative opioids from 84 to 8% and in terms of postoperative morphine consumption from 11 to 6% [63]. As previously stated, this multimodal approach has significantly reduced the risk of respiratory depression [64–66] and unexpected hospital admissions [67, 68]. The mostly used nonopioid adjuvant analgesic agents are dexmedetomidine, acetaminophen, and ketamine [64–66], as well as regional anesthesia techniques in the form of local infiltration, peripheral nerve blocks, and central neuraxial blocks [67, 68]. Also, the use of ultrasound-guided regional anesthesia techniques may increase its efficiency and safety in younger age. The potential neurotoxicity of general anesthesia should also be considered especially in younger children giving more priority to regional anesthesia [69]. The caudal block is one of the most used regional anesthesia techniques in children. This technique protects the children from perioperative pain with epidural administration of local anesthetics alone such as levobupivacaine, ropivacaine, and bupivacaine [70]. The perioperative analgesic efficacy of caudal blocks in children could be prolonged with addition of alpha-2 agonists (clonidine, dexmedetomidine), opioids, and ketamine as adjuvants to the local anesthetics [71, 72]. The combination of caudal block with periprocedural sedatives, such as dexmedetomidine and remifentanil, enables spontaneous breathing without airway manipulation and is associated with a lower aspiration risk, providing efficient postoperative analgesia in children who are undergoing lower abdominal/lower extremity surgery. That is why the incidence of respiratory complications is low and early ambulation in daily clinical practice is possible [73]. In the absence of studies referring to obese children, these facts could be recommended for this group of patients.

In ambulatory anesthesia, the airway is usually managed via an endotracheal tube or a laryngeal mask (LMA). Obese children are not an exception. Tracheal intubation is necessary for laparoscopic, neck, and ear-nose-throat procedures as a safer technique for controlling the airways. In case of a difficult intubation, the use of a fiberoptic bronchoscope, video laryngoscope, and a difficult airway cart should be considered before anesthesia induction. The insertion of a LMA in normal-weight children is associated with a lower incidence of PRAEs, such as laryngospasm, bronchospasm, and postoperative cough, compared to endotracheal tubes [74, 75]. On the other hand, it can increase the aspiration risk due to higher ventilation pressure caused by lower chest compliance of obese children. Encouraging results of LMA use in obese children undergoing minor surgery have been published recently. The insertion of LMA in obese children requires extended insertion time, higher peak airway pressure, lower pulmonary compliance, and postanesthesia care unit (PACU) stay time as well as less frequent postoperative coughing than in endotracheal intubation group [76]. This is supported by the fact that the choosing of size of LMA according to TBW significantly increases the oropharyngeal leak pressure and gives better ventilating conditions in overweight children [77]. We are unaware of any limitations or contraindications for the use of LMA in the pediatric population. Hence, in the future, the LMA could become the gold standard in airway maintenance for obese patients.

It is well known that children generally have higher oxygen consumption and a lower oxygen reserve developing thus hypoxemia faster than adults. Additionally, intraoperative desaturation is more common in obese children than in normal-weight children [48]. For this reason, the tolerance to apnea in these patients is lower, putting them at risk of developing rapid oxygen desaturation in the supine position during the induction of anesthesia. In order to prevent this, some authors advise adequate preoxygenation with 100% O_2_ for 3 min prior to induction of anesthesia [78]. Others suggest the use of 100% O_2_ for at least 5 min and continuous positive airway pressure (CPAP) of 10 cm H_2_O, or the ventilation via a face mask and the application of positive end-expiratory pressure of 10 cm H_2_O, and 25 degrees head-up position upon induction in order to facilitate the airway manoeuvers [79].

Mechanical ventilation should be adjusted to suit the pulmonary physiology of obese children. This is achieved by the application of a protective ventilation strategy that can maintain oxygenation, normocapnia and avoid lung damage. It involves the use of tidal volumes between 6 and 8 mL/kg IBW and optimal positive end-expiratory pressures to compensate for the lower functional residual capacity. In addition, lower oxygen levels are recommended in order to prevent reabsorption atelectasis and oxygen toxicity [80].

The use of nondepolarizing neuromuscular blocking drugs is feasible in ambulatory anesthesia, but residual blockade and its potential adverse effects are not completely known. Postoperative pulmonary complications were found to be more frequent when nondepolarizing neuromuscular blockers were used without neostigmine reversal. Based on these data, reversal of these drugs should be recommended for planned extubation [81], especially in OSA patients [82]. An improved reversion was seen with sugammadex in comparison to neostigmine due to minor postoperative pulmonary complications, but there is a need for more research in order to gather stronger evidence [83]. Removal of endotracheal tube or LMA can be done both in awake patients after full recovery of muscle strength or in deep anesthesia. Deep extubation may decrease the risk of overall airway complications including cough and desaturation; however, it may increase the airway obstruction as compared to awake extubation [84]. Before deep extubation, it is important to ensure unobstructed spontaneous breathing without excessive stimulation with airway suctioning. Also, the intraoperative administration of dexmedetomidine makes deep extubation easier and enables smooth postoperative recovery with minimal respiratory adverse events [85]. Despite this, it might be necessary to perform some interventions in obese children after extubation to maintain airway patency such as jaw thrust, placement of an oral or nasal airway, or placing the patient into the lateral or even prone position. The anesthesiologist should make the final decision about the most appropriate timing to extubate the patient.

### Postoperative care

Obese children have a significantly prolonged PACU stay and more frequent unexpected hospital admissions due to the development of postoperative respiratory complications as compared to normal weight children [86]. A higher incidence of postoperative respiratory depression has been associated with intraoperative administration of long-acting opioid drugs [87]. Postoperative respiratory depression induced by opioids can be avoided by the use of lower opioid doses intraoperatively, by the implementation of multimodal analgesia strategies [61, 88], and by administering naloxone [87]. This is especially important in the early postoperative period of obese children and children with OSA who are very prone to develop oxygen desaturation and hypoventilation in the recovery room [86, 87]. Therefore, close monitoring of oxygenation and ventilation with a pulse oximeter and capnography is mandatory for these patients [89]. Unavoidable postoperative airway interventions can be expected in children with OSA, who already require preoperative high flow nasal cannula, CPAP or bilevel positive airway pressure [90, 91]. This subgroup requires a prolonged stay in the PACU for overnight observation and monitoring until gain of full recovery and wakefulness. Therefore, these patients are considered ineligible for ambulatory anesthesia.

## Conclusion

Perioperative respiratory adverse events are common in pediatric patients during ambulatory anesthesia and are frequently associated with childhood obesity. Thorough preoperative assessment of risk factors is therefore mandatory, especially in obese children with respiratory comorbidities. Preoxygenation of obese children before induction to anesthesia, dose optimization of anesthetics, and analgesics combined with nonopioid analgesics, such as dexmedetomidine and regional anesthesia, should minimize the risk of postoperative respiratory depression. Additionally, noninvasive airway management, protective mechanical ventilation, and complete reversion of neuromuscular blockade and awake extubation are also suggested to reduce the frequency of PRAEs. Postoperative care and continuous monitoring of oxygenation and ventilation in this period are mandatory and can accelerate recovery after surgery in obese children.
